# Anti-CV2 Autoimmune Encephalitis With Parkinson-Like Symptoms and Bilateral Leukoencephalopathy—A Case Report

**DOI:** 10.3389/fneur.2019.01064

**Published:** 2019-10-09

**Authors:** Xuan Wu, Huayan Wang, Guorong Xu, Yu Lin

**Affiliations:** Department of Neurology, The First Affiliated Hospital of Fujian Medical University, Fuzhou, China

**Keywords:** paraneoplastic syndrome, leukoencephalopathy, encephalitis, Parkinson's disease, breast cancer

## Abstract

**Objective:** To present a patient with anti-CV2 autoimmune encephalitis admitted for Parkinson-like symptoms and bilateral leukoencephalopathy.

**Case report:** The patient was admitted for Parkinson-like symptoms combined with loss of taste. Serum anti-CV2 antibody was positive. Cranial magnetic resonance imaging revealed bilateral leukoencephalopathy. Breast cancer was detected by positron emission tomography (PET) and ultrasound. Immunotherapy was not performed. Modified radical mastectomy revealed a pT1cN0M0 breast cancer, positive for estrogen and progesterone receptors, and HER2 negative. The resting tremors disappeared by 1 week after surgery. The modified Rankin score (mRS) was four before surgery, and decreased to one at 9 months after surgery.

**Conclusion:** Anti-CV2 autoimmune encephalitis can present as Parkinsonism with bilateral leukoencephalopathy on MRI. PET scanning can be useful to reveal an occult cancer. Treatment of the cancer may improve the paraneoplastic neurological syndrome without the need of immunosuppressive therapy.

## Introduction

Autoimmune encephalitis is a central nervous system disease associated with autoantibodies against neuronal membranes and synaptic proteins, typically manifesting as seizure and mental and intellectual changes (1). The currently reported autoimmune encephalitis-related antibodies can be categorized as (2): 1) autoantibodies against neuronal surface receptors, including the most common anti-NMDAR, as well as anti-GABA_B_ receptor, anti-AMPAR, and anti-LGI1 antibodies; and 2) autoantibodies against neuronal intracellular antigens, including paraneoplastic syndrome-related antibodies such as anti-Hu, anti-Yo, anti-Ri, anti-Ma2, and anti-CV2. Among them, anti-CV2 autoimmune encephalitis is rare, and often manifests as choreic movements (3–6) and Parkinson-like symptoms (7). Unlike other types of autoimmune encephalitis, bilateral symmetric leukoencephalopathy revealed by imaging is rare.

In this report, we present the clinical characteristics, diagnosis, treatment, and follow-up of one patient with anti-CV2 autoimmune encephalitis whose initial clinical manifestations at admission were Parkinson-like symptoms. The imaging results showed bilateral symmetric extensive leukoencephalopathy.

## Case Report

The patient was a Chinese Han housewife of 52 years old, without contact with animals, with normal body mass index, and a place of residence without particularity. She was admitted on August 6, 2018 due to the main complaint of “slow movements and involuntary limb shaking for 3 months.” The patient had been suffering from slow movements for 3 months before admission, without obvious cause for physical retardation, and manifesting as slow walking speed and inflexibility, small split-steps, difficulty in turning around, and gradually worsening involuntary resting tremor. Those symptoms were accompanied by limb numbness and fatigue, dizziness, speech disfluency, small voice, loss of taste, and occasional urinary incontinence. On the other hand, there was no prodromal symptoms such as fever and headache, as well as no loss of consciousness and no limb convulsion. The patient had undergone routine cerebrospinal fluid examination in another hospital, and there was no obvious abnormality in cytology and biochemistry, and no pathogens. The patient was diagnosed with Parkinson's syndrome, and treated with benserazide and acupuncture. The symptoms did not improve and the patient was transferred to our hospital.

The patient had no previous history of exposure to toxic substances and drugs, no history of hypertension and diabetes, and no alcohol and tobacco consumption. The patient's father had a history of cancer. The physical examination at admission showed that the vital signs were normal, no cardiopulmonary abnormalities were observed, and no swelling of the superficial lymph nodes was observed. The patient was conscious and coherent, but with panic gait and few facial expressions. Limb muscle strength was normal, limb muscular tension was increased, and resting tremor of the limbs could be observed. Breast physical examination revealed no obvious mass. The modified Rankin score (mRS) was four. Blood routine, urine routine, fecal routine, liver and kidney functions, and electrolyte were all within the normal ranges. Serum anti-HIV antibody, antibodies against *Treponema pallidum*, hepatitis C antibody, and TORCH virus antibody were negative. Thyroid function, tumor markers, and immune indicators were normal. Urinary cadmium, arsenic, manganese, mercury, chromium, and strontium, and blood lead were normal. Serum anti-CV2 antibody was positive. Anti-Hu, anti-Ri, anti-Amphiphysin, anti-Tr, anti-GAD, anti-Yo, anti-Ma2, anti-NMDA receptor, anti-AMPA1 receptor, anti-AMPA2 receptor, anti-GABA_B_ receptor, anti-LGI1, and anti-CASPR2 antibodies were negative.

**Figure 1:**
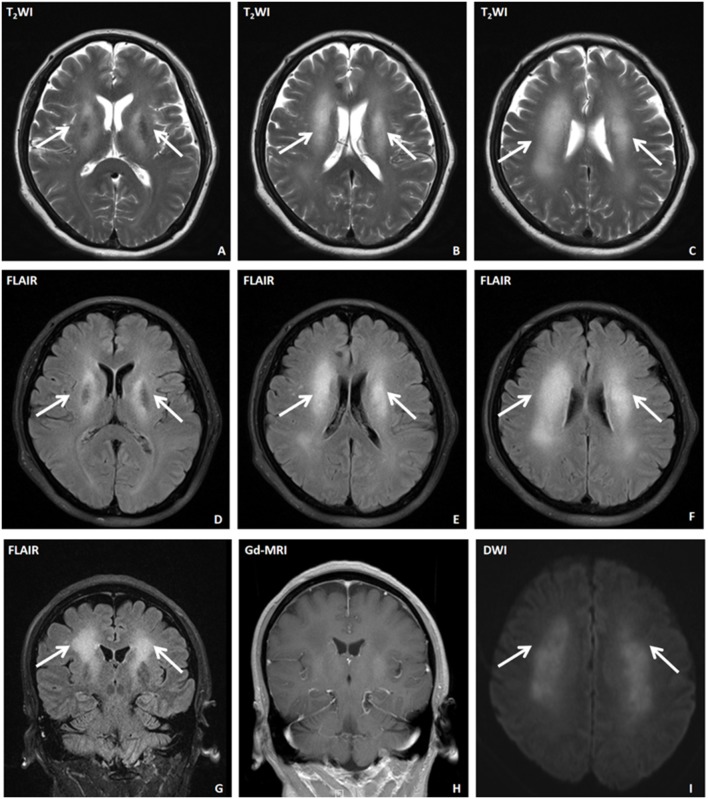
Cranial magnetic resonance imaging (MRI) of a patient with anti-CV2 autoimmune encephalitis. **(A–C)** T_2_WI axial images. **(D–F)** FLAIR axial images. **(G)** FLAIR coronal scan. **(H)** Enhanced MRI. **(I)** Diffusion-weighted MRI. The arrows indicate abnormal signals in the white matter area of the bilateral lateral ventricles and bilateral internal capsules. No significant enhancement was observed.

Lung computed tomography (CT) and dynamic electroencephalogram (EEG) were normal. Cranial magnetic resonance imaging (MRI) and contrast-enhanced MRI showed lesions in the white matter around the bilateral lateral ventricles and bilateral internal capsules, but there was no significant enhancement (Figure 1). Magnetic resonance spectroscopy (MRS) showed that the N-acetylaspartate (NAA) peak was decreased in the bilateral lateral ventricles; the Cho/NAA ratio was 2.49 (the left lateral ventricle was selected). Moreover, no Lip peak and lactic acid (lac) peak were observed. Whole-body positron emission tomography (PET)-CT showed that there were highly suspicious nodules in the upper quadrant of the left breast. Color Doppler ultrasound revealed a hypoechogenic nodule at the 10 o'clock position of the left breast. The nodule encompassed an area of about 1.1 × 0.6 cm; the boundaries of the nodule were unclear, and the internal echoes were uneven with rich strip-like blood flow signals. The BI-RADS classification was 4b.

At this point, the diagnoses was anti-CV2 autoimmune encephalitis and breast cancer. The patient was transferred to the Breast Surgery Department and underwent modified radical mastectomy under general anesthesia. Postoperative pathology revealed the presence of an invasive ductal carcinoma. Tumor heterogeneity was obvious under the fluorescence microscope. TNM staging was pT1cN0M0. The cancer was positive for estrogen receptors (ER; 90%), progesterone receptor (PR; 80%), HER2 (1+), hck (+), e-cadherin (+), and Ki-67 (8%), and negative for syn, cga, P63, and ck5/6. Fluorescence *in situ* hybridization (FISH) for HER2 was negative.

The resting limb tremors improved significantly by 1 week after operation. After surgery, the patient received oral toremifene. The patient was followed-up 9 months after surgery, and the results showed that limb muscle strength was normal. There was no involuntary limb shaking. There was only a slight speech disfluency and slightly slow movements. The mRS score was 1.

## Discussion

The patient was admitted with Parkinson-like symptoms and loss of taste. Serum anti-CV2 antibody was positive. Cranial magnetic resonance imaging revealed bilateral leukoencephalopathy. A breast cancer was detected by PET and ultrasound. The resting tremors disappeared by 1 week after surgery. The mRS was four before surgery, and decreased to one by 9 months after surgery. This case suggests that anti-CV2 autoimmune encephalitis can manifest as Parkinson-like symptoms and bilateral leukoencephalopathy. PET can be useful to reveal occult cancers in patients with suspected paraneoplastic syndrome. In the presence of white matter lesions only and a cancer, surgery to remove the cancer without immunosuppressive therapy may achieve a good prognosis.

Collapsing response mediator proteins-5 (CRMP-5) is a class of neuronal cytoplasmic proteins that are expressed in cerebral cortex, hippocampus, cerebellum, and thalamus of humans (8). Their genes are located on human chromosome 2 (9). CRMP-5 IgG is regarded as a neuronal autoantibody that may be a spontaneous immune response caused by small cell lung cancer and thymoma, and this antibody is not found in the blood of healthy subjects (9).

The CV2 antibody can specifically recognize CRMP-5, and anti-CV2 autoimmune encephalitis is a rare form of autoimmune encephalitis. At present, the reported clinical presentation of anti-CV2 autoimmune encephalitis includes chorea (3–6, 10), involuntary movements (11, 12), mental and behavioral abnormalities (4, 10), hypomnesia (5, 12, 13), convulsions (12, 13), and ataxia (3, 5, 13, 14). Moreover, there are rare cases with Parkinson-like symptoms (7), visual impairment (3, 14), myelitis (3, 10), and olfactory disorders (13, 15). The imaging features are mostly the involvement of striatum (3–5, 7, 10, 15), temporal lobes (5, 12, 13, 15), insular lobes (12, 13), and hippocampus (12, 16). There are also rare cases with involvement of the optic nerve (14), thalamus (17), and extensive white matter (4). In the patient presented here, the clinical symptoms were typical Parkinson symptoms. The patient was treated with benserazide in another hospital, but the symptoms did not improve. Imaging showed bilateral extensive leukoencephalopathy. The usual causes of leukoencephalopathy such as exposure to toxic substances and metabolic diseases were not observed. Therefore, the patient presented here is the first reported case of anti-CV2 autoimmune encephalitis with typical manifestations of Parkinson symptoms and bilateral extensive leukoencephalopathy.

The previously reported patients with anti-CV2 autoimmune encephalitis are all accompanied by malignant tumors, except for two cases reported by Vernino et al. (3) and Muehlschlegel et al. (4). Among the cancers reported to be associated with anti-CV2 autoimmune encephalitis, lung cancer, and thymic carcinoma are the most common, while testicular cancer, lymphoma, prostate cancer, and breast cancer were observed occasionally. It is worth noting that there is only one case of anti-CV2 autoimmune encephalitis associated with breast cancer, but the patient was also suffering from lung cancer (3). In the present case, lung CT showed no abnormality and whole-body PET-CT revealed no lesion except the breast lesion. Therefore, this patient is the first reported case of anti-CV2 autoimmune encephalitis who was only combined with breast cancer.

At present, immunosuppressive therapy is the main treatment for autoimmune encephalitis, but in the presence of a suspected paraneoplastic syndrome and a confirmed malignant tumor, standard cancer treatment (surgery, chemotherapy, radiotherapy, etc.) should be performed by the corresponding specialists (18). In the present case, hormones and other immunomodulatory treatments were not given and only cancer treatment was performed. After breast cancer surgery, the patient only had slightly speech disfluency and slow movements. The mRS score decreased from 4 to 1 without the use of immunosuppressive therapy, suggesting that immunosuppression may be not necessary for patients with autoimmune encephalitis combined with malignant tumors.

In conclusion, anti-CV2 autoimmune encephalitis can present as Parkinsonism with bilateral leukoencephalopathy on MRI. PET scanning can be useful to reveal an occult cancer. Treatment of the cancer may improve the paraneoplastic neurological syndrome without the need of immunosuppressive therapy.

## Data Availability Statement

All datasets generated for this study are included in the manuscript.

## Ethics Statement

The studies involving human participants were reviewed and approved by Branch for Medical Research and Clinical Technology Application, Ethics Committee of the First Affiliated Hospital of Fujian Medical University. The patients/participants provided their written informed consent to participate in this study.

## Author Contributions

GX was responsible for the data and the figures. XW was responsible for the intellectual content of the report. All authors cared for the patient and contributed to writing of the report.

### Conflict of Interest

The authors declare that the research was conducted in the absence of any commercial or financial relationships that could be construed as a potential conflict of interest.
